# The Relation Between Echocardiographic Epicardial Fat Thickness and CHA_2_DS_2_-VASc Score in Patients with Sinus Rhythm

**DOI:** 10.21470/1678-9741-2018-0230

**Published:** 2019

**Authors:** Fatih Aksoy, Serdar Guler, Fatih Kahraman, Tülay Oskay, Ercan Varol

**Affiliations:** 1 Department of Cardiology, Suleyman Demirel University, Medical School, Isparta, Turkey.

**Keywords:** Thromboembolism/Prevention & Control, Pericardium, Adipose Tissue, Risk Assessment

## Abstract

**Objective:**

To evaluate the predictive value of epicardial fat thickness (EFT) in CHA_2_DS_2_-VASc (congestive heart failure, hypertension, age ≥75 years, diabetes mellitus, previous stroke or transient ischemic attack, vascular disease, age 65-74 years, sex category) score risk groups.

**Methods:**

A total of 158 consecutive patients (75 females, 83 males, mean age 70.8±6.3 years) admitted routinely for cardiologic control were divided into two groups according to their CHA_2_DS_2_-VASc scores (scores 0 and 1 were regarded as low risk, and score ≥2 as high risk). One hundred twenty-five of 158 patients had a high-risk score.

**Results:**

Mean EFT was significantly higher in the high-risk group than in the low-risk group (4.34±0.62 *vs*. 5.37±1.0; *P*<0.001). EFT was positively correlated with CHA_2_DS_2_-VASc score (r=0.577, *P*<0.001). According to receiver operating characteristics (ROC) analysis, EFT value of 4.4 mm was found to be predictive of high risk in CHA_2_DS_2_-VASc score with 80% of sensitivity and 79% of specificity (C-statistic = 0.875, *P*<0.001, 95% confidence interval [CI] = 0.76-0.90). And according to multivariate logistic regression analysis, EFT was an independent predictor of high thromboembolic risk in terms of CHA_2_DS_2_-VASc score.

**Conclusion:**

Our findings suggest that echocardiographic EFT measurement could provide additional information on assessing cardiovascular risks, such as thromboembolic events, and individuals with increased EFT should receive more attention to reduce unfavorable cardiovascular risk factors and the development of future cardiovascular events.

**Table:** 

Abbreviations, acronyms & symbols				
ACEi	= Angiotensin-converting enzyme inhibitors		EFT	= Epicardial fat thickness
AF	= Atrial fibrillation		HDL	= High-density lipoprotein
ARB	= Angiotensin II receptor blockers		IQR	= Inter-quartile range
ASA	= Acetylsalicylic acid		IVS	= Interventricular septum
AUC	= Area under the curve		LA	= Left atrial/atrium
BMI	= Body mass index		LDL	= Low-density lipoprotein
CAD	= Coronary artery disease		LVEDD	= Left ventricular end diastolic diameter
CHA_2_DS_2_-VASc	= Congestive heart failure, hypertension, age ≥75years, diabetes mellitus, previous stroke or transient ischemic attack, vascular disease, age 65-74 years, sex category		LVESD	= Left ventricular end systolic diameter
CI	= Confidence interval		LVPW	= Left ventricular posterior wall
CT	= Computed tomography		NT-proBNP	= N-terminal pro b-type natriuretic peptide
DBP	= Diastolic blood pressure		NVAF	= Non-valvular atrial fibrillation
EAT	= Epicardial adipose tissue		OA/NOA	= Oral anticoagulant/New oral anticoagulant
EDTA	= Ethylenediaminetetraacetic acid		OR	= Odds ratio
EF	= Ejection fraction		ROC	= Receiver operating characteristics
			SBP	= Systolic blood pressure
			TIA	= Transient ischemic attack

## INTRODUCTION

Ischemic stroke is a leading cause of death and long-term disability worldwide^[1]^. Control of risk factors is of particular importance for the prevention of cerebrovascular diseases. It is possible to stop progression or prevent these diseases by elimination or modification of modifiable risk factors in the light of treatment goals.

Epicardial adipose tissue (EAT), located between the myocardium and visceral pericardium, has emerged as an important cardiovascular risk predictor, in view of producing and releasing several adipocytokines^[2,3]^. The importance of epicardial fat thickness (EFT) has been shown in recent years. Increased EFT is associated with hypertension, insulin resistance, and thromboembolic processes such as stroke and acute coronary syndrome^[4-6]^.

The CHA_2_DS_2_-VASc risk score is a cheap and easy scoring system which is calculated by assigning 1 point for each: congestive heart failure (ejection fraction [EF] < 40%), hypertension, age between 65 and 74 years, diabetes mellitus, vascular disease (myocardial infarction or peripheral arterial disease), and female sex; and 2 points for: a history of stroke or transient ischemic attack (TIA) and age > 75 years. The CHA_2_DS_2_-VASc risk score is used to predict the thromboembolism risk in non-valvular atrial fibrillation (NVAF) patients^[7]^.

The present study aimed to determine whether EFT is more closely associated with high-risk patients according to the CHA_2_DS_2_-VASc risk score.

## METHODS

The 158 consecutive patients (75 females, 83 males, mean age 70.8±6.3 years) admitted to the outpatient clinic of the Suleyman Demirel University Hospital, Department of Cardiology, and referred to our echocardiography laboratory due to suspicion of heart disease between June 2014 and May 2015 were enrolled in this prospective study. All patients underwent medical history assessment, physical examination, anthropometric measurements, electrocardiogram, and echocardiographic evaluation. The study was approved by the institutional ethics committee and all patients gave their informed consent. Exclusion criteria were pericardial effusion, poor echocardiographic window, history of chronic renal and liver disease, moderate to severe mitral and aortic regurgitation, moderate to severe mitral and aortic stenosis, malignancy, systemic or pulmonary embolism, chronic hematological diseases, acute or chronic inflammatory disease, autoimmune disease, hyperparathyroidism, hypercalcemia, hyperphosphatemia, and a prosthetic valve. According to CHA_2_DS_2_-VASc score, patients were divided into two groups: scores 0 and 1 were regarded as low risk, and score ≥2 as high risk.

### Echocardiography

**Fig. 1:**
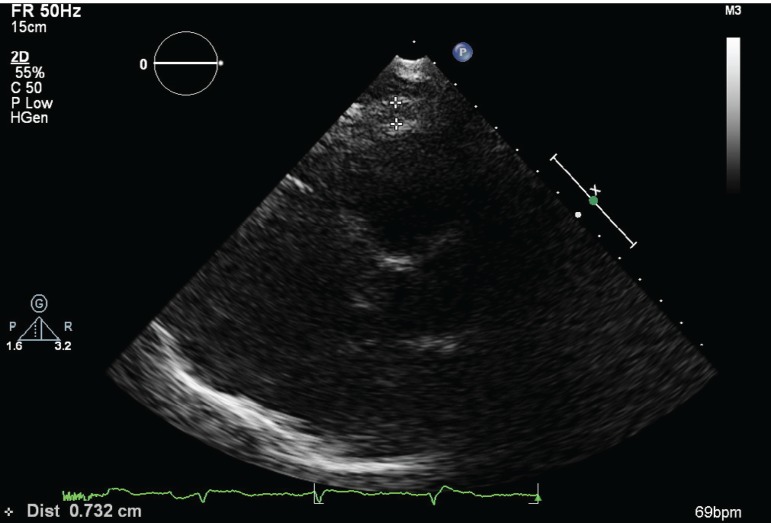
Measurement of epicardial fat thickness by echocardiography.

The M-mode, two-dimensional, and Doppler echocardiographic examinations were obtained by an ultrasound machine (Philips iE 33 xMatrix) to assess left atrial (LA) diameter, interventricular septum (IVS) thickness, left ventricular posterior wall (LVPW) thickness, left ventricular end diastolic diameter (LVEDD), left ventricular end systolic diameter (LVESD), and left ventricular EF. LA and left ventricular dimensions and left ventricular EF were measured by M-mode echocardiography in the parasternal long axis view by using the American Echocardiography Society M-mode technique^[8]^. The presence of mitral and aortic insufficiency was evaluated by Doppler color flow mapping. EFT was identiﬁed echocardiographically as the echo-free space between the outer wall of the myocardium and the visceral layer of pericardium. EFT was measured at the point on the free wall of the right ventricle along the midline of the ultrasound beam, perpendicular to the aortic annulus at the end of systole^[4]^ (Figure 1). As Iacobellis et al.^[4]^ suggested, epicardial fat is best measured at end-systole, because it is compressed during diastole. The average value of three cardiac cycles was determined as EFT.

### Blood Sampling

Blood samples were drawn from the antecubital vein by careful venipuncture in a 21 G sterile syringe without stasis at 08.00-10.00 AM after a fasting period of 12 h. Glucose, creatinine, and lipid profiles were determined by standard methods. Hemogram parameters were measured in a blood sample collected in dipotassium ethylenediaminetetraacetic acid (EDTA) tubes (Vacuette). An automatic blood counter (Beckman-Coulter Co, Miami, FL, USA) was used for whole blood counts.

### Statistical Analysis

SPSS software package program, version 16.0, was used in this study's statistical analyses. Categorical variables were expressed as frequency (%) and compared using the χ^2^ test. A Kolmogorov-Smirnov test was used to test the distribution of numeric variables, and those with normal distribution were expressed as mean ± standard deviation and were compared using the Student's *t*-test. Data without normal distribution were expressed as median (inter-quartile range [IQR] of 25%-75% percentiles) and were compared using the Mann-Whitney U test. In all statistical analyses, *P* values <0.05 were considered as statistically significant. Univariate analysis of binary logistic regression was carried out to identify which factors were associated with high risk according to CHA_2_DS_2_-VASc risk score. After including each of these potential confounding factors, backward conditional binary logistic regression analysis was performed to estimate the odds ratio (OR) and 95% confidence interval (CI) for high risk according to CHA_2_DS_2_-VASc risk score. Receiver operating characteristics (ROC) curve analysis was used to analyze the prognostic value of EFT for high risk according to CHA_2_DS_2_-VASc risk score. C-Statistic (area under the curve [AUC]) was presented as a unified estimate of sensitivity and specificity. According to the cut-off value that was obtained by a ROC curve analysis, the study population could be segregated into two groups, as low risk and high risk. The correlations between CHA_2_DS_2_-VASc risk score, EFT, and other clinical, laboratory, and echocardiographic parameters were performed with Pearson and Spearman correlation analysis when appropriate.

## RESULTS

**Fig. 2:**
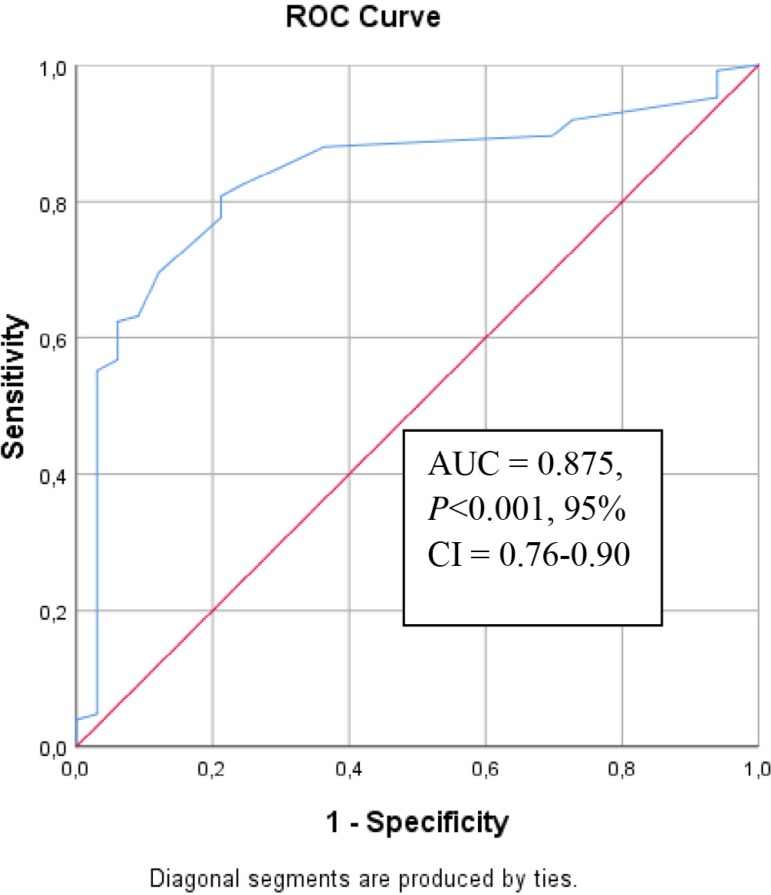
Receiver operating characteristics (ROC) curve with calculated area under the curve (AUC) and optimal cut-off point for epicardial fat thickness to identify the presence of high risk of CHA2DS2-VASc (congestive heart failure, hypertension, age ≥75 years, diabetes mellitus, previous stroke or transient ischemic attack, vascular disease, age 65-74 years, sex category) score. CI=confidence interval

Baseline clinical features of the study population were summarized in Table 1. Age, female gender, hypertension, and diabetes mellitus were seen more often in high CHA_2_DS_2_-VASc score group than in low CHA_2_DS_2_-VASc score group. Only β-blocker and clopidogrel usages were significantly higher in the high CHA_2_DS_2_-VASc score group. Laboratory findings of the study population were summarized in Table 2. There was no statistically significant difference between the two groups except for fasting glucose (*P*=0.04). Cholesterol levels were similar between high- and low-risk groups according to CHA_2_DS_2_-VASc score. Echocardiographic findings of the study population were summarized in Table 3. LA diameter was significantly higher in patients with high-risk score than in low-risk score subjects in terms of CHA_2_DS_2_-VASc score (33±5.6 *vs*. 36±4.3 mm, respectively; *P*<0.001). IVS thickness was significantly higher in patients with high risk than in low-risk subjects (10±1.0 *vs*. 11±1.4 mm, respectively; *P*<0.001). LVEDD was significantly higher in patients with high risk than in low-risk subjects (44±1.7 *vs*. 45±3.7 mm, respectively; *P*<0.001). LVESD was significantly higher in patients with high risk than in low-risk subjects (27±1.7 *vs*. 28±3.3 mm, respectively; P<0.001). EFT was significantly higher in patients with high risk than in low-risk subjects (4.34±0.62 *vs*. 5.37±1.0 mm, respectively; *P*<0.001). Correlation analysis between EFT and CHA_2_DS_2_-VASc score with other clinical and echocardiographic parameters was shown in Table 4. EFT was positively correlated with CHA_2_DS_2_-VASc score (r=0.577, *P*<0.001). Also, EFT was positively correlated with age (r=0.520, *P*<0,001), LA (r=0.264, *P*<0.001), IVS (r=356, *P*<0.001), LVESD (r=0.262, *P*=0.011), and aorta diameter (r=0.22, *P*<0.001). Negative correlation was found between EFT and left ventricular EF (r=-0.199, *P*=0.012). CHA_2_DS_2_-VASc score was positively correlated with age (r=0.578, *P*<0,001), LA (r=0.235, *P*=0.003), IVS (r=386, *P*<0.001), LVESD (r=0.337, *P*<0.001), and aorta diameter (r=0.229, *P*=0.004). Negative correlation was found between CHA_2_DS_2_-VASc score and left ventricular EF (r=-0.154, *P*=0.05). Univariate and multivariate regression analyses results were shown in Table 5. Older age, LA diameter, aorta diameter, left ventricular EF, IVS diameter, and EFT achieved statistical significance in the univariate logistic analysis. Then, multivariate analysis was carried out with these variables; age and EFT were found to be independent predictors of high risk for CHA_2_DS_2_-VASc classification. According to ROC analysis, EFT value of 4.4 mm was predictive of high risk of CHA_2_DS_2_-VASc score with 80% of sensitivity and 79% of specificity (C-statistic = 0.875, *P*<0.001, 95 % CI= 0.76-0.90; Figure 2).

**Table 1:** Baseline clinical features of the study population.

Parameter	Low CHA_2_DS_2_-VASc score (n=33)	High CHA_2_DS_2_-VASc score (n=125)	*P*
Age, years	64±4.1	72±5.9	<0.001
Female gender, n (%)	9 (26)	66 (53)	<0.001
Hypertension, n (%)	5 (14)	85 (70)	<0.001
Diabetes mellitus, n (%)	4 (11)	58 (46)	<0.001
Hyperlipidemia, n (%)	18 (52)	56 (47)	0.359
Smoking, n (%)	7 (20)	53 (42)	0.014
CAD, n (%)	-	31 (24)	<0.001
Stroke/TIA, n (%)	-	11 (8)	0.069
BMI (kg/m^2^)	29±3.5	30±7.0	0.209
SBP (mmHg)	109±9	122±17	<0.001
DBP (mmHg)	73±6	76±10	0.109
Heart rate (beat/min)	70±12	71±13	0.623
*ASA*, n (%)	17 (51)	49 (39)	0.183
Clopidogrel, n (%)	-	20 (16)	<0.001
*OA/NOA*, n (%)	-	8 (6)	0.146
Statin, n (%)	8 (24)	32 (25)	0.535
ACEi, n (%)	4 (12)	34 (27)	0.053
ARB, n (%)	5 (15)	32 (25)	0.159
β-blocker, n (%)	6 (17)	55 (44)	<0.001

ACEi=angiotensin-converting enzyme inhibitors; ARB=angiotensin II receptor blockers; ASA=Acetylsalicylic acid; BMI=body mass index; CAD=coronary artery disease; CHA_2_DS_2_-VASc=congestive heart failure, hypertension, age ≥75 years, diabetes mellitus, previous stroke or transient ischemic attack, vascular disease, age 65-74 years, sex category; DBP=diastolic blood pressure; OA/NOA=Oral anticoagulant/New oral anticoagulant; SBP=systolic blood pressure; TIA=transient ischemic attack

**Table 2:** Laboratory findings of the study population.

Parameter	Low CHA_2_DS_2_-VASc score (n=33)	High CHA_2_DS_2_-VASc score (n=125)	*P*
Hemoglobin, g/L	12.1±1.1	11.9±1.0	0.201
Platelet count (x 10^3^/µL)	238±58	245±71	0.603
White blood cell count (x 10^3^/µL)	8251±2344	7730±2400	0.264
Fasting glucose, mg/dL	111±34	128±62	0.04
HDL-cholesterol, mg/dL	45±15	47±12	0.444
LDL-cholesterol, mg/dL	114±43	111±36	0.763
Triglycerides, mg/dL	178±131	148±83	0.109

CHA_2_DS_2_-VASc=congestive heart failure, hypertension, age ≥75 years, diabetes mellitus, previous stroke or transient ischemic attack, vascular disease, age 65-74 years, sex category; HDL=high-density lipoprotein; LDL=low-density lipoprotein

**Table 3:** Echocardiographic findings of the study population.

Parameter	Low CHA_2_DS_2_-VASc score (n=33)	High CHA_2_DS_2_-VASc score (n=125)	*P*
Left ventricular EF, %	60± 1.9	59± 3.7	<0.001
Aorta (mm)	24± 1.5	25± 2.4	<0.001
LA (mm)	33± 5.6	36± 4.3	<0.001
IVS (mm)	10± 1.0	11± 1.4	<0.001
LVPW (mm)	9.3± .05	10± 0.8	<0.001
LVESD (mm)	27± 1.7	28± 3.3	<0.001
LVEDD (mm)	44± 1.7	45± 3.2	<0.001
EFT (mm)	4.34± 0.62	5.37± 1.0	<0.001

CHA_2_DS_2_-VASc=congestive heart failure, hypertension, age ≥75 years, diabetes mellitus, previous stroke or transient ischemic attack, vascular disease, age 65-74 years, sex category; EF=ejection fraction; EFT=epicardial fat thickness; IVS=interventricular septum; LA=left atrium; LVEDD=left ventricular end diastolic diameter; LVESD=left ventricular end systolic diameter; LVPW=left ventricular posterior wall

**Table 4:** Clinical and echocardiographic parameters showing the significant correlation with EFT and CHA_2_DS_2_-VASc score.

	With EFT		With CHA_2_DS_2_-VASc score	
	r	*P*	r	*P*
Age	0.520	<0.001	0.578	<0.001
LA	0.264	0.001	0.235	0.003
IVS	0.356	<0.001	0.386	<0.001
LVESD	0.262	0.011	0.337	<0.001
LVEDD	0.188	0.018	0.202	0.011
LVEF	-0.199	0.012	-0.154	0.05
Aorta	0.22	0.004	0.229	0.004
Waist circumference	0.184	0.02	0.151	0.05
BMI	0.156	<0.001	0.172	0.03
CHA_2_DS_2_-VASc score	0.577	<0.001	0.577	<0.001

BMI=body mass index; CHA_2_DS_2_-VASc=congestive heart failure, hypertension, age ≥75 years, diabetes mellitus, previous stroke or transient ischemic attack, vascular disease, age 65-74 years, sex category; LVEF=left ventricular ejection fraction; EFT=epicardial fat thickness; IVS=interventricular septum; LA=left atrium; LVEDD=left ventricular end diastolic diameter; LVESD=left ventricular end systolic diameter

**Table 5:** Predictors of CHA_2_DS_2_-VASc risk classification in univariate and multivariate analyses.

	OR	(95 % CI)	*P* value	OR	(95 % CI)	*P* value
Age (years)	1.318	1.17-1.47	<0.001	1.270	1.11-1.44	<0.001
Epicardial tissue thickness	7.01	2.89-16.9	<0.001	4.0	1.61-10.28	0.003
Left ventricular ejection fraction (%)	0.876	0.76-0.997	0.045			
Left atrial length (mm)	1.161	1.05-1.27	<0.001			
Aorta diameter	1.341	1.10-1.62	<0.001			
IVS	1.770	1.27-2.45	<0.001			

CI=confidence interval; IVS=interventricular septum; OR=odds ratio

## DISCUSSION

In the present study, we examined EFT in patients with high- and low-risk CHA_2_DS_2_-VASc scores. We found out that EFT was significantly higher in patients with high CHA_2_DS_2_-VASc score than in those with low CHA_2_DS_2_-VASc score and that EFT was positively correlated with CHA_2_DS_2_-VASc scores.

EAT is a true visceral fat tissue, deposited around the heart and particularly around the subepicardial coronary vessels. EAT is a complex organ, mainly composed of adipocytes, but it also includes a neuronal network, stromavascular, immune and inflammatory cells, all nourished by a rich microcirculation^[2,3,9]^.

EFT is associated with thromboembolic diseases, including cardiovascular and neurovascular diseases^[5,6]^. Akil et al.^[6]^ showed that EFT was significantly higher in patients with ischemic stroke than in healthy controls. Akdag et al.^[10]^ investigated the association of EFT, inflammatory, and thrombosis parameters with CHA_2_DS_2_-VASc score in NVAF patients. They determined that EFT, inflammatory, and thrombosis parameters were associated with the thromboembolic risk exhibited by CHA_2_DS_2_-VASc score in NVAF patients. In our study, we investigated the association of EFT with CHA_2_DS_2_-VASc score in patients with sinus rhythm. Our results were similar with those from that study. EFT was significantly higher in high CHA_2_DS_2_-VASc score than in low CHA_2_DS_2_-VASc score among patients with sinus rhythm. EAT is considered an endocrine and metabolically active organ. It is a source of several bioactive molecules that can influence the myocardium and coronary arteries^[11]^. Epicardial fat expresses and secretes a number of cytokines, pro- and anti-inflammatory adipokines, vasoactive factors, and growth factors^[11,12]^. Accordingly, inflammation appears to play an important role in thromboembolic events, such as acute coronary syndrome and stroke^[13,14]^. As a result, increased inflammatory mediators from EAT may have an important role in the pathogenesis of stroke and atherosclerosis. As also mentioned before, EAT is strongly associated with the pathogenesis of atherosclerosis due to sharing the same risk factors. In the present study, we have shown that high thromboembolic risk according to CHA_2_DS_2_-VASc score was positively correlated with EFT.

It has been shown that EAT is related to cardiovascular risk factors^[15]^. The studies using echocardiography to measure EFT on the right ventricle showed relations with waist circumference and left ventricular measurements^[15-18]^. Our findings were similar to these studies. We reported a strong relationship between EAT and age, diabetes mellitus, and hypertension. CHA_2_DS_2_-VASc score includes these risk factors. Similarly, Cetin et al.^[19]^ reported a significant association between EFT and type 2 diabetic subjects with subclinical atherosclerosis. Dogan et al.^[20]^ showed that in patients with newly diagnosed hypertension, increased EFT was significantly linked to impaired aortic elastic properties. Iacobellis et al.^[17]^ showed a correlation between cholesterol levels and EFT. However, the relationship between EFT and CHA_2_DS_2_-VASc score was independent of cholesterol levels in our study.

In the echocardiographic evaluation, EFT, LA length, aorta diameter, and IVS length were correlated with CHA_2_DS_2_-VASc score and they are independent risk factors for high thromboembolic risk in CHA_2_DS_2_-VASc score, based on a multivariate analysis. Accordingly, Altun et al.^[21]^ have shown a significant association between EFT, N-terminal pro b-type natriuretic peptide (NT-proBNP) levels, and arterial dysfunction in patients who had sustained acute ischemic stroke.

Stroke is one of the most important causes of death and long-term disability. Control of the risk factors can prevent the development of stroke^[1]^. Recent studies have shown a relation between EFT and stroke^[6,22]^. Akil et al.^[6]^ demonstrated for the first time the association between EFT and cerebral ischemic stroke.

As mentioned before, EAT has the same blood supply as the adjacent myocardium and also shown paracrine functions. This causes risk for cardiac structures due to local pathogenic inflammatory effects^[2,11,12]^. A computed tomography (CT) evaluation from the Framingham Heart study showed that pericardial fat volume could predict atrial fibrillation (AF) risk independently of other measurements of adiposity^[23]^. Tsao et al.^[22]^ showed that periatrial EAT was increased and was correlated with atrial dysfunction in patients with AF-related stroke.

EAT, a metabolically active tissue can induce fibrotic changes on the atrial myocardium by releasing proinflammatory cytokines and adipo-fibrokines. EAT can be an infiltrated adipocyte on the atrial myocardium. This can cause blockage of local conduction and promote the micro-reentry circuit. As a result, the occurrence of AF increases. Two potential mechanisms can be proposed for this association: firstly, the actions of proinflammatory cytokines and adipo-fibrokines released from EAT, such as activin A, adiponectin, and resistin, which can induce fibrotic changes on the atrial myocardium^[2,4,11]^; and secondly, adipocyte infiltration on the atrial myocardium, which can cause blockage of local conduction and promote the micro-reentry circuit; and potential modulations of the autonomic nervous system by the ganglionic plexus within the EAT, which may influence the occurrence of AF. In this study, we comprehensively assessed the relationship between the CHA_2_DS_2_-VASc score with EFT around the right ventricle. They were independently associated with each other, based on a multivariate analysis. Consequently, we can say that EFT is a risk indicator for stroke.

### Study Limitations

The relatively limited number of patients could limit the strength of the results and the conclusion obtained from this study. Echocardiographic EAT is a linear measurement, and thus it may not assess the total epicardial fat volume that varies at several myocardial locations. As a result of EAT being a metabolically active tissue, inflammatory cytokines and inflammatory markers could be investigated in future studies.

## CONCLUSION

In conclusion, our findings suggest that echocardiographic EFT measurement could provide additional information on assessing cardiovascular risks, such as thromboembolic events, and individuals with increasing EFT should receive more attention to reduce unfavorable cardiovascular risk factors and the development of future cardiovascular events.

**Table:** 

Authors' roles & responsibilities	
FA	Substantial contributions to the conception or design of the work; or the acquisition, analysis, or interpretation of data for the work; final approval of the version to be published
SG	Substantial contributions to the conception or design of the work; or the acquisition, analysis, or interpretation of data for the work; final approval of the version to be published
FK	Drafting the work or revising it critically for important intellectual content; final approval of the version to be published
TO	Drafting the work or revising it critically for important intellectual content; agreement to be accountable for all aspects of the work in ensuring that questions related to the accuracy or integrity of any part of the work are appropriately investigated and resolved; final approval of the version to be published
EV	Drafting the work or revising it critically for important intellectual content; final approval of the version to be published
